# Comparative transcriptomic profiling of field-grown cassava genotypes across season transitions

**DOI:** 10.1038/s41597-025-06119-w

**Published:** 2025-11-21

**Authors:** Mariam Webber-Birungi, Joseph Enye Iboyi, Erik Alexandersson, Andreas Gisel, Livia Stavolone

**Affiliations:** 1 International Institute of Tropical Agriculture (IITA), Ibadan, Nigeria; 2 Department of Plant Breeding, Swedish University of Agricultural Sciences (SLU), Sundsvägen 10, SE-234 22, Lomma, Sweden; 3 Institute of Biomedical Technology, Italian National Research Council (CNR), Bari, Italy; 4 Institute for Sustainable Plant Protection, Italian National Research Council (CNR), Bari, Italy; 5 Present Address: Corteva Agriscience, 8325 NW 62nd Ave, Johnston, Iowa 50131 USA; 6 Present Address: Novo Nordisk Fonden, Tuborg Havnevej 19, 2900 Hellerup, Denmark

**Keywords:** Drought, Plant molecular biology

## Abstract

Cassava (*Manihot esculenta*, Crantz) is a perennial crop cultivated in tropical and subtropical areas. In its cultivation cycle, it encounters environmental stresses related to changes in temperature and water fluctuations during seasonal transitions. We profiled the transcriptomes of four field-grown genotypes to investigate the molecular basis of adaptation to season transitions. 3’mRNA-seq libraries were prepared from samples collected from storage roots to capture gene expression changes associated with shifts from rainy to dry and dry to rainy seasons. Reproducibility and variability within the dataset were evaluated using correlation analysis and principal component analysis, providing confidence in data quality and consistency across samples. The usability of these data was proved by differential expression analysis during the rainy-to-dry and dry-to-rainy transitions, and by functional enrichment analysis. The detailed information of the experimental environmental conditions and of the workflow from planting to final DEGs analysis provided, make this dataset a useful resource for future research on plant responses to environmental fluctuations and to identify candidate genes for crop improvement strategies for climate-resilient varieties.

## Background & Summary

Cassava (*Manihot esculenta*, Crantz), a crucial crop in tropical and subtropical regions, is the third most important source of calories in the tropics, providing a reliable carbohydrate source for hundreds of millions of people worldwide^1^. Its global production exceeds 300 million metric tons annually, with the majority cultivated in Africa, Asia, and Latin America^2,3^.

Cassava’s global significance stems from its remarkable adaptability to diverse environmental conditions. This resilient crop thrives in diverse climate conditions, from humid tropics to arid and semi-arid regions. Its resilience to challenging growing conditions, including poor soils, pests, and diseases, supports its role as a dependable crop for smallholder farmers in areas with limited access to modern agricultural inputs^4^. These capabilities become increasingly crucial as agriculture confronts escalating challenges due to climate change, where rising temperatures and more frequent extreme weather events threaten crop productivity worldwide^5^.

As a tropical root crop cultivated in an annual cycle, cassava experiences distinct seasonal transitions, from rainy to dry periods and back to rainy conditions, each imposing unique biotic and abiotic stresses that impact its growth, physiology, and overall resilience^6^. During rainy seasons, cassava benefits from ample moisture, promoting nutrient uptake and growth^7^. However, the dry season brings reduced soil moisture, increased temperatures, and solar radiation, inducing drought-like conditions that challenge cassava’s physiological and cellular mechanisms^8^. To withstand these seasonal shifts, cassava activates a variety of adaptive responses, including reducing leaf area, changing stomatal conductance to conserve water, modifying root architecture to enhance water uptake, and altering gene expression to regulate stress-responsive proteins^9–11^.

Investigating the transcriptomic changes associated with these seasonal patterns is critical for understanding the mechanisms underlying cassava’s resilience, with general implications for crop improvement under fluctuating climate conditions. While previous studies have considered cassava’s drought responses under controlled conditions, understanding its adaptation to natural seasonal transitions remains limited. Controlled environments often fail to capture the complexity of field environments, where multiple stressors interact dynamically.

Based on this foundation, this study adopts a broader approach by profiling transcriptomes of field-grown cassava across natural seasonal transitions within a single growing season. We applied 3’mRNA Illumina sequencing, reducing sequencing depth and costs and allowing accurate quantification of gene expression. The depth and quality of obtained data confirms and encourages the use of such technology for comparative transcriptome analysis.

The discovery of shared gene expression patterns across four cassava genotypes, with diverse growth habits, disease resistance, and yield performance (Table S1), indicates common adaptive strategies to seasonal environmental changes, providing broadly applicable insights into its molecular responses useful for breeders and biotechnologists. Linking transcription factors to their target genes further validates the dataset. Unlike controlled studies, this field-based approach moves beyond controlled stress conditions, offering a dynamic perspective on cassava’s natural adaptation to environmental changes.

## Methods

### Plant materials and experimental conditions

**Fig. 1:**
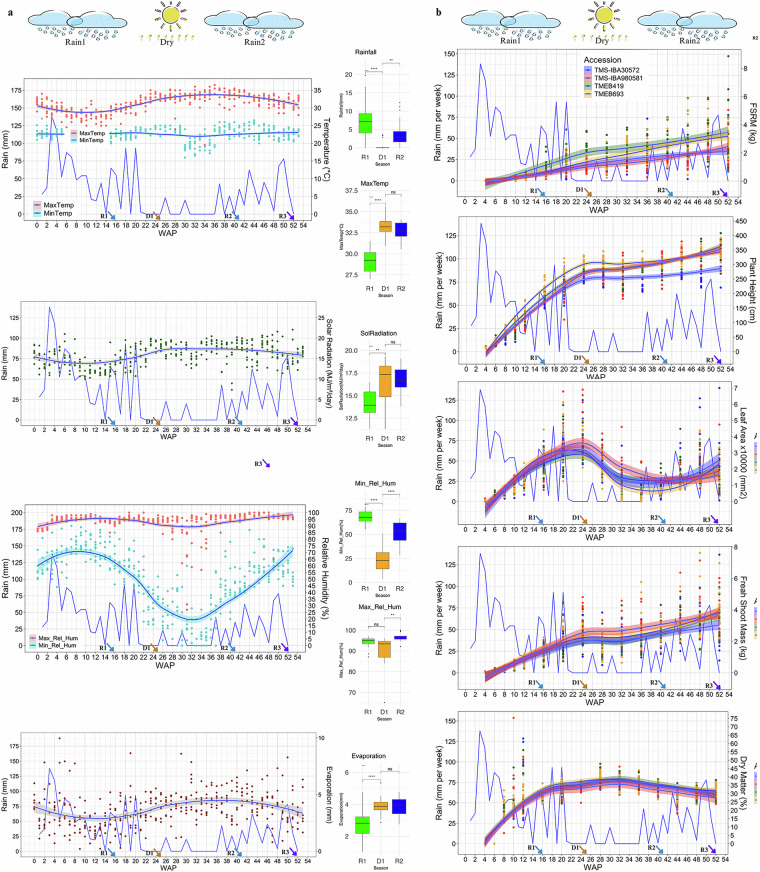
Weather parameters and cassava agronomical trait trends across seasonal transitions. (**a**) Weekly average of daily measurements of weather parameter: temperature (°C), relative humidity (%), solar radiation (MJ/m²), and evaporation (mm). Alongside box plots summarizing these averages. The rainfall graph (mm) indicates the cumulative rain per week. (**b**) Trends of agronomical traits, including fresh storage root mass (FSRM, kg), plant height (PH, cm), leaf area (LA, mm²), shoot mass (kg), and dry matter content (DM, %) across the seasonal transitions. Data represent the average of 12 replicate for each time point and each of the four genotypes (TMEB419, TMEB693, TMS30572, TMS980581). WAP, week after planting. Arrows indicate key sampling points at 16 WAP (R1, Rain1), 25 WAP, (D1, Dry), 41 WAP, (R2, Rain2), and 52 WAP, (R3, Rain3).

For this study, four cassava genotypes, TMEB419, TMEB693, TMS-IBA30572, and TMS-IBA980581 (Table S1), were cultivated in the field, in a randomized complete block design, at IITA Forest Reserve, Westbank (7.490708^0^ N, 3.883849^0^ E). Their growth parameters: plant height (PH), leaf area (LA), fresh shoot mass (FSM), fresh storage root mass (FSRM), and dry matter (DM), were monitored bi-weekly for the first 3 months (until bulking of storage roots) and then monthly during seasonal transitions (Growth parameters). Daily weather monitored at the IITA weather station throughout the growing season characterized the environmental conditions experienced by the crop and defined the seasonal transitions (Weather data). These sub-tropical seasonal transitions, from the first rainy season to the following dry season and to the second rainy season, coincide with pronounced variations in environmental conditions, including temperature, rainfall, solar radiation, evaporation, and relative humidity that can shape transcriptional reprogramming can shape transcriptional reprogramming, and were, therefore, monitored throughout the field experiment (Fig. 1a).

To expand the possibility to use these transcriptomic data in future analysis, we also measured agronomical traits indicative of cassava’s physiological and developmental responses to seasonal transitions. (Fig. 1b).

### RNA extraction and sequencing

For transcriptomic analysis, storage root of the four field-grown genotypes were sampled in triplicate at four time points: R1, 16 WAP during Rain1; D1, 25 WAP during Dry, R2, 41 WAP at start of the Rain2, and R3, at 52 WAP during the second rains (Fig. 1).

Destructive samples comprised a mixture of three different roots for each plant replicate. Total RNA was extracted from storage root tissue of the four above-mentioned genotypes (3 independent biological replicates per genotype) by combining cetyltrimethylammonium bromide (CTAB)-extraction method and spin-column based purification^12^. The purified RNA was resuspended in RNase-free water, and RNA quality was preliminarily assessed using a NANODROP 8000 spectrophotometer (Thermo Scientific, Waltham, MA USA). RNA samples with OD260/280 and OD260/230 values ranging between 1.9 and 2.2 were selected for further analysis. The RNA integrity was then assessed by gel electrophoresis using 1.2% agarose gel with 1 × Tris/Borate/EDTA buffer (Sigma-Aldrich, St. Louis, MO, USA), at 80 V for 40 minutes. Based on this analysis, samples were selected for 3’mRNA-Seq library preparation according to the manufacturer’s protocol (Quant Seq, Lexogene, Vienna, Austria). Sequencing was performed on an Illumina NextSeq 500 platform, generating 75-bp single-end reads.

### RNAseq processing, quality control, and mapping

Raw reads were mapped against the reference genome of *Manihot esculenta* v8.0 in Phytozome^13^ using the splicing site-sensitive mapping tool STAR v.2.4.1c and STAR index, including the corresponding GFF annotation file. STAR parameters were set to eliminate low-quality mappings and include: i) only reads aligning with matching read fragments longer than 50% of the full read length, ii) only matching read fragments with less than 4 mismatches (6% of the total read length and iii) only alignments with a score higher than 66% of the full length.

### Pre-processing and quantification of transcripts

An in-house-developed Perl script (https://github.com/gisels4/3primeTag/) was used to parse the STAR mapping file and create clusters of overlapping read hits. The number of reads in each cluster was counted, reflecting the expression level for each gene (cluster). Within each cluster, the script searched for reads with a polyA tail that had been soft clipped by the STAR aligner. The poly A tail is an indication that the cluster indeed represents the end of a transcript. However, we expect that some clusters, although representing a transcript, is missing reads with a poly A tail. Therefore, the script compares the biological replicates and selects only clusters with at least two poly A tails containing reads and at least one cluster within the three biological replicates should contain these two reads with a poly A tail. The output of this script was a count matrix for each annotated gene across all samples and replicates.

### Normalization of gene expression data

Gene-level read counts were analyzed using R v4.4.1 and the DESeq 2 package v1.44.0^14^. Raw count matrices were first filtered to exclude low-expression genes (defined as having <5 counts in fewer than 3 samples) to reduce noise and improve statistical power. Filtered counts were then normalized using DESeq 2’s median-of-ratios method, which corrects for sequencing depth and compositional differences across libraries by estimating size factors per sample^15^. To stabilize variance across a wide range of mean expression values, we applied DESeq 2’s variance-stabilizing transformation (VST). The VST approach mitigates the heteroscedasticity inherent in count data, producing a log2-like transformed matrix appropriate for unsupervised analyses^14^. This transformed matrix was used for all downstream clustering, dimensionality reduction, and correlation analyses.

### Principle component analysis

Unsupervised principal component analysis (PCA) was performed using base R on the VST-transformed expression matrix, with centering and scaling enabled. The first two principal components (PC1 and PC2) captured 68.7% and 4.3% of the total variance, respectively. These components effectively separated samples according to genotype and physiological condition, reflecting dominant biological signals. To interrogate treatment-specific transcriptional shifts, separate PCA analyses were conducted on sample subsets (e.g., rain-to-dry and dry-to-rain comparisons), highlighting condition-dependent expression patterns.

### Sample correlation and clustering

Sample-level consistency was assessed through Pearson correlation analysis of all pairwise sample comparisons using the VST data. Correlation matrices were visualized using the pheatmap package v1.0.12^16^, with hierarchical clustering applied to both rows and columns. Sample annotations by genotype and treatment were overlaid to validate grouping structure. The observed clustering confirmed strong within-group correlations and reproducibility of biological replicates. No technical outliers were detected.

### Differential expression analysis

Gene expression data from three biological replicates per genotype at each time point (R1, D1, R2) were analyzed using DESeq 2^14^. Comparisons were made across seasonal transitions (R1 vs D1 and D1 vs R2) and among genotypes. Genes with a log2 fold change (LFC) greater than 0.5 or less than −0.5 and a false discovery rate (FDR) < 5% were designated as significantly differentially expressed genes (DEG).

### Functional annotation of genes

Gene sets of shared DEG were annotated using the Database for Annotation Visualization and Integrated Discovery (DAVID)^17,18^ with Ensembl gene IDs as input (Gene annotation). Genes labeled as “unknown” were mapped by extracting unspliced transcript sequences from the *Manihot esculenta* version 8.0 genome using the BioMart (Phytozome). The sequences were queried using the PlantRegMap ID mapping tool^19,20^, leveraging reciprocal best hits (RBHs) to link query sequences with annotated IDs. For uncharacterized genes, transcription factor (TF) prediction, TF target identification, and cis-regulatory element analysis were performed to explore potential functional roles.

### Gene ontology and pathway enrichment analysis

Gene ontology (GO) enrichment analysis was conducted using the PlantRegMap GO enrichment tool, incorporating annotations from TAIR 10, UniProt-GOA, InterProScan, and RBHs with. *Manihot esculenta* genes as background set. Significant GO terms were identified using Fisher’s exact test in TopGO (p-value ≤ 0.05; GO terms). Pathway enrichment analysis of shared DEGs (intersecting all genotypes per transition) was performed using ShinyGO V0.77^21^. Genes were queried against the PlantGSAD database^22^ which integrates KEGG pathways including cellular and viral pathways^23,24^ (KEGG pathways).

### Genes re-regulation analysis

DEG that were common across all genotypes (shared DEG) in both transitions were analyzed for re-regulation. A gene was considered re-regulated if it met one of the following criteria: (i) it was a sigDEG in one transition (log2 fold change ≥ 0.5, FDR < 5%) but not in the other, or (ii) its expression pattern was reversed between transitions (i.e., shifting from upregulation to downregulation or vice versa).

### Transcription factors identification and enrichment

TFs were predicted from input nucleic acid sequences of shared gene sets using PlantTFDB v5.0, following established family assignment rules^19,25^. Briefly, input sequences were first processed with EST Scan 3.0 to identify coding regions (CDS) and they were then translated into proteins sequences. TF families were assigned based on the best hit in *Arabidopsis thaliana*. Enrichment analysis was performed using PlantRegMap^20^, which integrates data from literature, ChiP-seq experiments, and motifs-binding analyses. Fisher’s exact test was used to assess significant enrichment of TF families among target genes at p-value ≤ 0.05.

## Technical Validation

### Quality control

We sequenced, on average, 13.6 million 3’mRNA-Seq reads per sample, generated a total of 49.86 Gb across 48 libraries from four cassava genotypes (TMEB419, TMEB693, TMS30572, TMS980581) sampled at four seasonal transition time points (R1, D1, R2, R3), sequencing yielded between ~7.7 and 24.8 million reads per library. Read quality exceeded a score per position of 28 for nearly all libraries (except one sample), with average scores between 28–35.

### Mapping quality

**Fig. 2:**
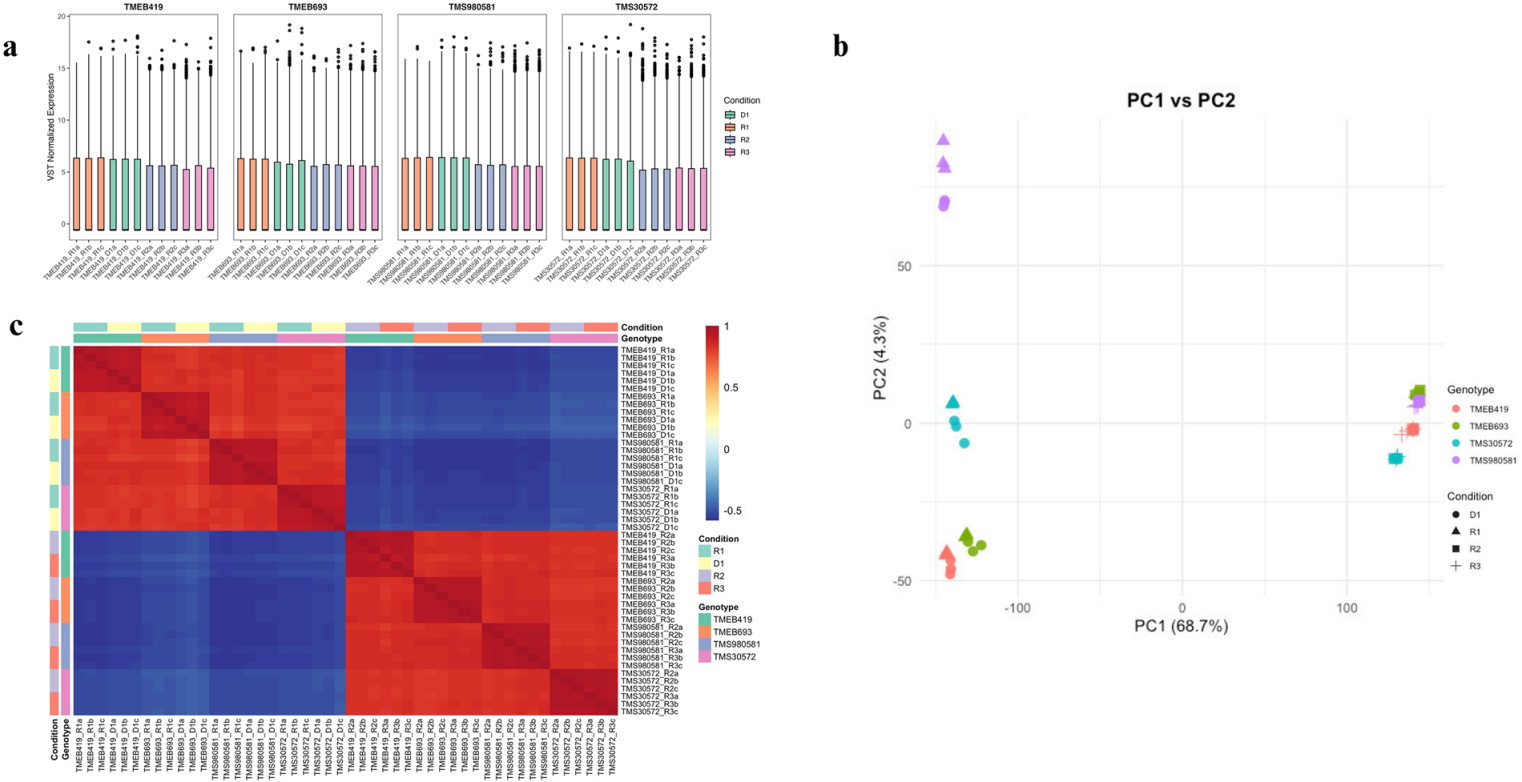
Quality assessment and exploratory analysis of RNA-seq data. (**a**) Box plots of variance-stabilized transformed (VST) expression values across all expressed genes for each RNA-seq sample. Boxes represent the interquartile range (IQR), with the central line indicating the median. Samples are grouped by genotype and condition, demonstrating consistent normalization, minimal technical variability, and absence of extreme outliers, supporting data quality and comparability for downstream analyses. (**b**) Principal component analysis (PCA) plots based on VST-normalized counts. Left panel: PC1 (68.7% variance) versus PC2 (4.3%), capturing major variance and separating samples by genotype and condition and demonstrating the low variability between biological replicates. (**c**) Heatmap of pairwise Pearson correlations between samples, computed from VST-normalized gene expression. Color scale ranges from low (blue) to high (red) correlation. High intra-group correlations and distinct inter-group patterns confirm replicate consistency and transcriptional divergence driven by genotype and condition.

66.5–94.4% (average 86.6%) mapped uniquely to the *Manihot esculenta* version 8.0 reference genome, with a mean mapped read length of ~70 nt (from 76 bp raw reads), a per base mismatch rate of 0.9–1.3%, and 4.8–16.8% mapping to multiple loci. These values indicate good mapping quality and dataset reliability. Consistent expression distribution among replicates further supported data quality (Fig. 2a). Summary statistics by genotype and time point are shown in Table 1, with full replicate level details in Supplementary Fig S2. PCA of full variance-stabilized expression matrix showed a clear structure, with PC1 and PC2 explaining 68.7% and 4.3% of the total variance, respectively (Fig. 2b). A complementary heatmap supports the PCA results, demonstrating reproducible biological signal across replicates and grouping patterns consistent with experimental design (Fig. 2c).

**Table 1:** Summary of sequencing and mapping statistics.

Genotype	Time point	Samples (n)	Input reads (million)	Uniquely mapped (%)	Multi-mapped (%)	Avg. mapped length (nt)	Mismatch rate (%)
**TMEB419**	R1	3	9.5–14.9 (mean 11.7)	81.9–92.6 (mean 87.9)	5.8–9.1 (mean 7.6)	69.2–71.7	1.14–1.26
	D1	3	13.1–22.5 (mean 18.0)	81.8–87.5 (mean 83.8)	7.9–9.5 (mean 8.8)	68.5–69.8	1.26–1.32
	R2	3	10.4–12.8 (mean 11.9)	79.9–86.9 (mean 84.1)	9.4–15.7 (mean 12.3)	66.5–69.5	1.07–1.15
	R3	3	7.8–12.7 (mean 9.8)	77.3–88.9 (mean 81.9)	9.1–15.6 (mean 13.4)	65.5–69.8	1.04–1.21
**TMEB693**	R1	3	11.3–24.8 (mean 16.6)	85.7–87.3 (mean 86.7)	8.3–8.6 (mean 8.4)	70.0–70.6	1.16–1.18
	D1	3	8.4–14.2 (mean 11.7)	66.5–91.1 (mean 80.1)	6.1–9.2 (mean 7.8)	67.0–69.6	1.18–1.32
	R2	3	10.3–15.2 (mean 13.1)	75.1–94.4 (mean 85.3)	5.1–16.8 (mean 10.7)	66.1–71.6	0.92–1.18
	R3	3	9.1–10.0 (mean 9.6)	80.5–89.1 (mean 85.7)	8.9–15.3 (mean 11.4)	66.7–69.6	1.03–1.12
**TMS980581**	R1	3	11.4–19.6 (mean 15.0)	84.1–91.8 (mean 87.7)	6.4–8.9 (mean 7.9)	69.4–71.4	1.11–1.23
	D1	3	13.3–17.6 (mean 16.1)	85.9–89.5 (mean 88.0)	7.0–8.2 (mean 7.6)	69.9–70.9	1.14–1.21
	R2	3	10.0–15.7 (mean 12.9)	85.7–94.4 (mean 89.7)	4.8–10.4 (mean 7.7)	69.1–72.3	0.98–1.02
	R3	3	10.9–11.7 (mean 11.2)	89.8–91.1 (mean 90.5)	7.2–8.2 (mean 7.6)	69.8–71.0	1.02–1.03
**TMS30572**	R1	3	18.9–22.2 (mean 20.3)	87.6–89.9 (mean 88.9)	7.3–7.9 (mean 7.6)	70.7–70.9	1.23–1.28
	D1	3	11.0–23.9 (mean 17.6)	81.4–90.0 (mean 86.8)	6.9–7.7 (mean 7.4)	69.9–70.5	1.25–1.27
	R2	3	9.4–12.1 (mean 10.8)	89.1–92.8 (mean 90.9)	5.4–7.6 (mean 6.7)	69.9–72.0	1.07–1.20
	R3	3	10.0–15.2 (mean 12.4)	88.8–90.4 (mean 89.8)	8.0 (consistent)	70.1–70.2	1.09–1.12

RNA-seq libraries (n = 48) from four cassava genotypes (TMEB419, TMEB693, TMS980581, and TMS30572) sampled during four seasonal time points (R1, D1, R2, and R3). Values represent the range and mean across three biological replicates per genotype × time point. Shown are input reads (in millions), uniquely mapped reads (%), multi-mapped reads (%), average mapped read length (nt), and per-base mismatch rate (%).

### Stress-responsive gene dataset

**Fig. 3:**
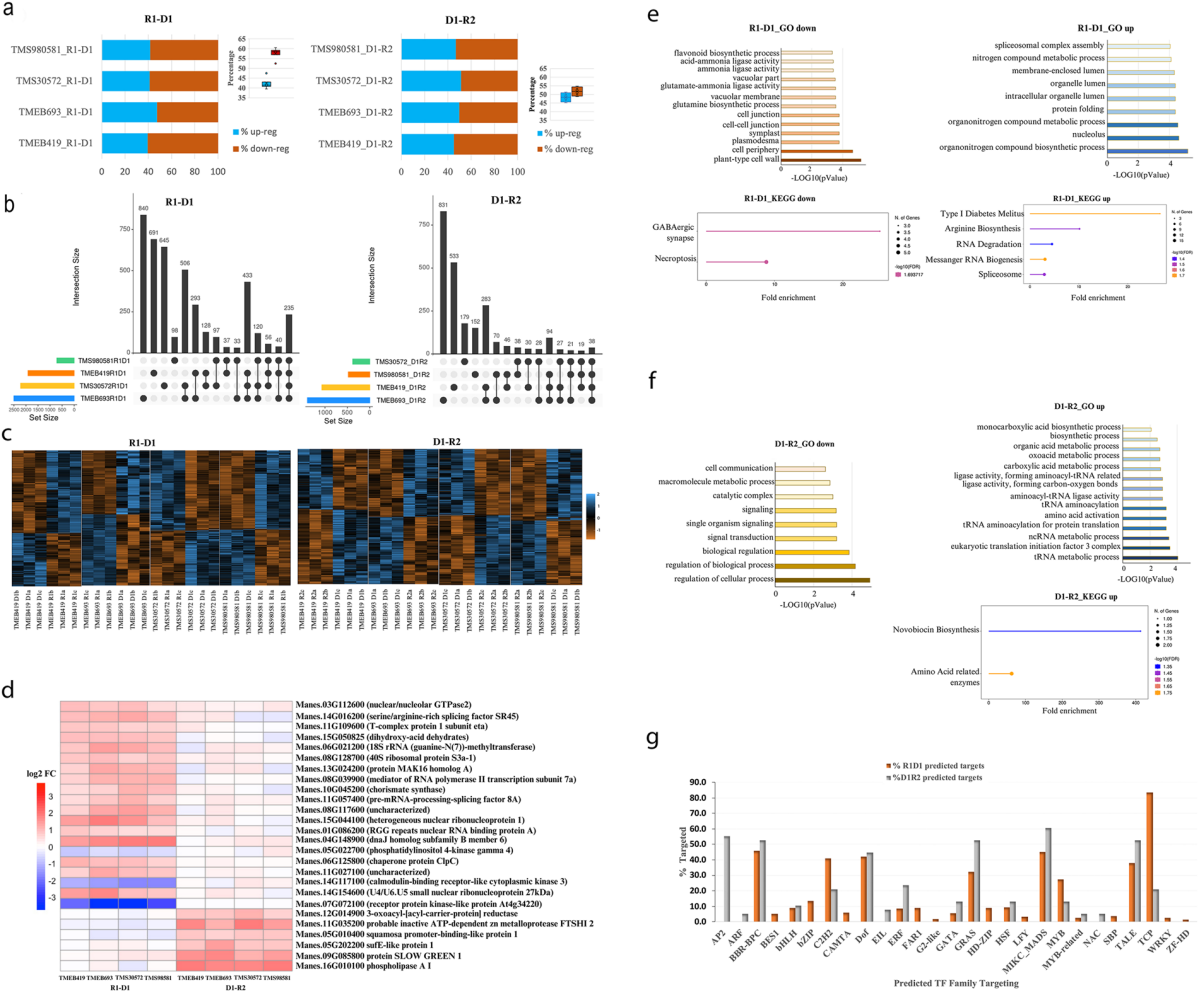
Gene dataset functional validation (**a**) Percentage proportions of the up- (blue) and down- (brown) regulated genes, and in-set the proportion range among genotypes. (**b**) UpSet plot matrices showing unique genes (single dot) and shared genes (linked dots) within genotypes. Color bars on a scale next to plots indicate gene set size. (**c**) Hierarchically clustered heatmaps per genotype for all sigDEGs revealing up- (blue) and down- (brown) regulated gene expression differences in R1 vs D1, and D1 vs R2 comparisons. (**d**) Shared genes that were differentially expressed in genotypes below or above the threshold (logFC 0.44, FDR = 5%) and changed to above or below the threshold in the subsequent transition comparison were considered relatively re-regulated (heatmap scale = Log2 Fold Change). (**e**) Enriched GO terms and KEGG pathways in shared genes R1-D1 downregulated (left) and R1-D1 upregulated (right). (**f**) Enriched GO terms and KEGG pathways in shared genes D1-R2 downregulated (left) and D1-R2 upregulated (right). (**g**) Target TF families and percentage (%) proportion of predicted targets in shared SigDEGs of R1-D1 (Orange) and D1-R2 (Grey) transitions.

To confirm the relevance and usability of our dataset, differential expression analysis across seasonal transitions identified extensive transcriptional reprogramming across all genotypes during seasonal transitions as visualised in the heatmaps, the percent proportion of the up- and down-regulated genes, and the UpSet plot matrices of unique and shared genes between genotypes (Fig. 3). During R1-D1, 1,837 DEG were identified, of which 52–60% were downregulated (Table 2). In D1-R2, 830 DEG were found, with 45–51% upregulated. These DEG included well-characterized stress-responsive genes, such as aquaporin PIP2-1 (reduced water loss), osmotic-like proteins (osmotic balance), chaperone protein dnaJ, **(**protein stabilization), and galacturonosyltransferase 8 (GAUT8), essential for cell wall remodeling. We further identified 26 re-regulated genes that returned to baseline expression with the return of the rain season (D1-R2) after stress removal consistent with transcriptional resetting during rehydration (Fig. 3d). Their dynamic expression aligns with known stress-recovery mechanisms, confirming the dataset’s utility for studying transcriptional resetting. Enrichment analysis confirmed the involvement of GO and the KEGG pathways associated with abiotic stress and recovery during seasonal transitions (Fig. 3e, f). TF family enrichment revealed both shared (HSF, TCP, bZIP, MYB, Dof) as well as transition specific regulators such as bZIP, BES1, and HD-ZIP in R1D1, and AP2, ARF, and NAC in D1-R2, suggesting seasonal-specific regulatory roles (Fig. 3g).

**Table 2:** Number of significantly Differentially Expressed Genes (DEG) identified at a >0.5 logfold change (LFC) threshold and <5% false discovery rate in each genotype and season comparison.

Season transition	Genotype	DEG		
		Up regulated	Downregulated	Total
R1-D1	TMEB419	756	1157	1913
	TMEB693	1188	1312	2500
	TMS30572	915	1305	2220
	TMS980581	298	418	716
	*mean*	*789*	*1048*	*1837*
D1-R2	TMEB419	484	586	1070
	TMEB693	694	698	1392
	TMS30572	195	185	380
	TMS980581	224	254	478
	*mean*	*399*	*431*	*830*

Our dataset analysis reveals a dynamic blueprint of cassava molecular resilience and can be useful for the study of stress response to climate changes. This stress response comprises a cascade of fundamental elements, including the perception of stress, signal transduction, activation of stress responses or gene regulation/alteration, and subsequent adaptation/acclimation that we have summarized in Supplementary Fig. S1 to facilitate further use of the dataset. Finally, we provide a table of genes and pathways to be considered as potential actionable targets for drought mitigation and recovery strategies (Actionable targets). Experimental validation of these key genes and pathways, combined with field-based studies, will be crucial for translating these into strategies for breeding climate-resilient cassava varieties.

## Data Records

High-throughput sequencing data supporting this study’s findings have been deposited at ENA European Nucleotide Archive^26^.

In addition, plant growth parameters, weather data, gene annotations, GO terms, KEGG pathways, transcription factors (TF) targets, and actionable target genes are uploaded to Zenodo^27^ (10.5281/zenodo.15545011).

## Supplementary information

Supplementary information

## Data Availability

High-throughput sequencing data supporting this study’s is accessible without restriction in the ENA European Nucleotide Archive under the following link: https://identifiers.org/ena.embl:PRJEB79515 (2024). All metadata datasheets: plant growth parameters, weather data, gene annotations, GO terms, KEGG pathways, transcription factors (TF) targets, and actionable target genes are accessible in the Zenodo repository under the following link: (10.5281/zenodo.15545011).

## Code availability

Software and their versions used for RNA-seq analysis were described in Methods. Custom codes are available at https://github.com/gisels4/3primeTag.

## References

[1] Hood, A. S. C. *et al*. A systematic map of cassava farming practices and their agricultural and environmental impacts using new ontologies: Agri-ontologies 1.0. *Ecological Solutions and Evidence* **4** (2023).

[2] Hashid, A. & Almaqtari, F. A. The Impact of Artificial Intelligence and Industry 4.0 on Transforming Accounting and Auditing Practices. *Journal of Open Innovation: Technology, Market, and Complexity* **10**, 100218 (2024).

[3] FAO. *FAOSTAT Analytica Brief Series No. 60. Rome*. 10.4060/cc3751en (2022).

[4] Mupakati, T. & Tanyanyiwa, V. I. Cassava production as a climate change adaptation strategy in Chilonga Ward, Chiredzi District, Zimbabwe. *Jàmbá: Journal of Disaster Risk Studies* **9** (2017). 10.4102/jamba.v9i1.348 PMC6014075 29955331

[5] CIAT. *Resilient and Ready: Cassava Stands up to Climate Change*. https://ciat.cgiar.org/news/resilient-and-ready-cassava-stands-up-to-climate-change/ (2020).

[6] El-Sharkawy, M. A. Cassava biology and physiology. *Plant Mol Biol* **56**, 481–501 (2004). 15669146 10.1007/s11103-005-2270-7

[7] Alves, A. A. C. Cassava botany and physiology. *Cassava: biology, production and utilization* 67–89, 10.1079/9780851995243.0067 (2002).

[8] Mittler, R. Oxidative stress, antioxidants and stress tolerance. *Trends Plant Sci* **7**, 405–410 (2002). 12234732 10.1016/s1360-1385(02)02312-9

[9] Ike, I. F. & Thurtell, G. W. Osmotic adjustment in indoor grown cassava in response to water stress. *Physiol Plant* **52**, 257–262 (1981).

[10] El-Sharkaway, M. A. & Cock, J. H. Water Use Efficiency of Cassava. I. Effects of Air Humidity and Water Stress on Stomatal Conductance and Gas Exchange1. *Crop Sci* **24**, 497 (1984).

[11] Okogbenin, E. *et al*. Phenotypic approaches to drought in cassava: review. *Front Physiol* **4** (2013). 10.3389/fphys.2013.00093 PMC3650755 23717282

[12] Carluccio, A. V. *et al*. Set up from the beginning: The origin and early development of cassava storage roots. *Plant Cell Environ* **45**, 1779–1795 (2022). 35229892 10.1111/pce.14300 PMC9314696

[13] Goodstein, D. M. *et al*. Phytozome: a comparative platform for green plant genomics. *Nucleic Acids Res* **40**, D1178–D1186 (2011). 22110026 10.1093/nar/gkr944 PMC3245001

[14] Love, M. I., Huber, W. & Anders, S. Moderated estimation of fold change and dispersion for RNA-seq data with DESeq 2. *Genome Biol* **15**, 550 (2014). 25516281 10.1186/s13059-014-0550-8 PMC4302049

[15] Anders, S. & Huber, W. Differential expression analysis for sequence count data. *Genome Biol* **11** (2010). 10.1186/gb-2010-11-10-r106 PMC3218662 20979621

[16] Kolde, R. pheatmap: Pretty Heatmaps. R package version 1.0.12. Preprint at https://CRAN.R-project.org/package=pheatmap (2019).

[17] Hosack, D. A., Dennis, G., Sherman, B. T., Lane, H. C. & Lempicki, R. A. Identifying biological themes within lists of genes with EASE. *Genome Biol* **4** (2003). 10.1186/gb-2003-4-10-r70 PMC328459 14519205

[18] Sherman, B. T. *et al*. DAVID: a web server for functional enrichment analysis and functional annotation of gene lists (2021 update). *Nucleic Acids Res* **50** (2022). 10.1093/nar/gkac194 PMC9252805 35325185

[19] Jin, J., Zhang, H., Kong, L., Gao, G. & Luo, J. PlantTFDB 3.0: a portal for the functional and evolutionary study of plant transcription factors. *Nucleic Acids Res* **42**, D1182–D1187 (2013). 24174544 10.1093/nar/gkt1016 PMC3965000

[20] Tian, F., Yang, D.-C., Meng, Y.-Q., Jin, J. & Gao, G. PlantRegMap: charting functional regulatory maps in plants. *Nucleic Acids Res* **48** (2019). 10.1093/nar/gkz1020 PMC7145545 31701126

[21] Ge, S. X., Jung, D. & Yao, R. ShinyGO: a graphical gene-set enrichment tool for animals and plants. *Bioinformatics* **36**, 2628–2629 (2019). 10.1093/bioinformatics/btz931 PMC7178415 31882993

[22] Ma, X. *et al*. PlantGSAD: A comprehensive gene set annotation database for plant species. *Nucleic Acids Res* **50** (2022). 10.1093/nar/gkab794 PMC8728169 34534340

[23] Kanehisa, M. & Goto, S. KEGG: Kyoto Encyclopedia of Genes and Genomes. *Nucleic Acids Research* **28**, 10.1093/nar/28.1.27 (2000). 10.1093/nar/28.1.27 PMC102409 10592173

[24] Kanehisa, M., Furumichi, M., Sato, Y., Ishiguro-Watanabe, M. & Tanabe, M. KEGG: integrating viruses and cellular organisms. *Nucleic Acids Res* **49**, D545–D551 (2020). 10.1093/nar/gkaa970 PMC7779016 33125081

[25] Jin, J. *et al*. PlantTFDB 4.0: toward a central hub for transcription factors and regulatory interactions in plants. *Nucleic Acids Res* **45**, D1040–D1045 (2016). 27924042 10.1093/nar/gkw982 PMC5210657

[26] *ENA European Nucleotide Archive* https://identifiers.org/ena.embl:PRJEB79515 (2024). 10.1093/nar/gkae975 PMC11701661 39558171

[27] Gisel, A., T Webber-Birungi, M., & Stavolone, L. Comparative transcriptomic profiling of field-grown cassava genotypes across season transitions [Data set]. *Zenodo*. 10.5281/zenodo.15545011 (2025) 10.1038/s41597-025-06119-w PMC12639095 41271766

