# Supplementary material for: Comparative transcriptomic profiling of field-grown cassava genotypes across season transitions
Source: Sci Data. 2025 Nov 21;12:1858. doi: 10.1038/s41597-025-06119-w (PMC12639095; doi:10.1038/s41597-025-06119-w)
Supplement: Supplementary file 1 — Supplementary information [file 41597_2025_6119_MOESM1_ESM.pdf]

Table S1. Features of the sample genotypes (Source: cassavabase.org)

Features	TMEB419	TMEB693	TMS-IBA980581	TMS-IBA30572
Staygreen visual scale (1-9)	3.1 (1.5)	4.8 (2.8)	3.1 (1.3)	3.7 (1.5)
Level of branching	2.7	17.7	1.5 (1.1)	3.5 (8.6)
Growth habit	Straight	Straight	Straight	Multi-branching
Cassava Mosaic Disease (1-5)	1.2 (0.5)<br>Resistant	1.1 (0.2)<br>Resistance	1. (0.1)<br>Resistant	1.9 (0.9)<br>Moderate
Incidence of Cassava bacterial blight (1-5)	1.7 (0.9)	0.8 (0.3)	0.0	1.4 (6.3)
Leaf retention (1-9)	5.5 (6.5)	6.8 (7.0)	3.6 (1.6)	5.3 (4.5)
Severity of Green mites (1-5)	2.9 (1.4)	3.8 (1.0)	3.0 (1.2)	3.7 (0.9)
Average Yield t/ha (fresh/dry)	23.8 (59.0)/8.8 (24.2)	14.0 (10.4)/4.9 (3.7)	23.6 (20.1)/8.2 (8.2)	15.8 (13.8)/5.4 (5.8)

Table S2. Detailed sequencing and mapping statistics for all cassava RNA-seq libraries. Read quality and alignment metrics for each of the 48 RNA-seq libraries used in this study. Shown are total input reads, uniquely mapped reads (number and %), average mapped length (nt), mismatch rate per base (%), number of reads mapped to multiple loci, and % of multi-mapped reads. These values are reported for each biological replicate within each genotype × time point.

Sample	Description (Genotype_time point)	Number of input reads	Uniquely mapped reads number	%Uniquely mapped reads	Average mapped length (raw read 76nts)	Mismatch rate per base, %	Number of reads mapped to multiple loci	% of reads mapped to multiple loci
A4_1	TMEB419_R1	9511733	8496878	89,3	69,96	1,19%	739371	7,8
A4_3	TMEB419_R1	14885527	12184341	81,9	69,21	1,26%	1355122	9,1
A4_5	TMEB419_R1	10326656	9561118	92,6	71,66	1,14%	603670	5,8
A4_7	TMEB419_D1	13116761	10783023	82,2	68,62	1,32%	1196818	9,1
A4_9	TMEB419_D1	18851294	15429273	81,8	68,51	1,26%	1791683	9,5
A4_11	TMEB419_D1	22534879	19719575	87,5	69,76	1,26%	1778507	7,9
A4_13	TMEB419_R2	10365373	8279850	79,9	66,51	1,15%	1630372	15,7
A4_15	TMEB419_R2	12400188	10779457	86,9	69,53	1,09%	1165911	9,4
A4_17	TMEB419_R2	12838194	10973242	85,5	68,67	1,07%	1499814	11,7
A4_19	TMEB419_R3	10004861	7735981	77,3	65,51	1,21%	1563157	15,6
A4_21	TMEB419_R3	12718301	11301272	88,9	69,78	1,04%	1159182	9,1
A4_23	TMEB419_R3	7761259	6162324	79,4	66,67	1,14%	1206929	15,6
B4_1	TMEB693_R1	11302788	9872205	87,3	70,12	1,17%	944877	8,4
B4_3	TMEB693_R1	24770068	21222796	85,7	69,96	1,18%	2136292	8,6
B4_5	TMEB693_R1	13565745	11825638	87,2	70,58	1,16%	1130298	8,3
B4_7	TMEB693_D1	8412559	7667539	91,1	69,55	1,30%	510435	6,1
B4_9	TMEB693_D1	12400434	8244367	66,5	66,99	1,32%	1138572	9,2
B4_11	TMEB693_D1	14219114	11778069	82,8	69,56	1,18%	1161964	8,2
B4_13	TMEB693_R2	10265707	7710354	75,1	66,07	1,18%	1729515	16,8
B4_15	TMEB693_R2	15172889	13125949	86,5	68,91	1,02%	1529895	10,1
B4_17	TMEB693_R2	13081146	12354305	94,4	71,55	0,92%	666506	5,1
B4_19	TMEB693_R3	9560808	8520622	89,1	69,63	1,03%	848501	8,9
B4_21	TMEB693_R3	10038094	8777417	87,4	69,32	1,03%	1017786	10,1
B4_23	TMEB693_R3	9096184	7319122	80,5	66,74	1,12%	1387642	15,3
D4_1	TMS980581_R1	11421416	9600912	84,1	69,42	1,23%	1015942	8,9
D4_3	TMS980581_R1	14103973	12298756	87,2	70,31	1,20%	1187645	8,4
D4_5	TMS980581_R1	19555645	17951189	91,8	71,36	1,11%	1259572	6,4
D4_7	TMS980581_D1	17603336	15127636	85,9	70,04	1,21%	1443713	8,2
D4_9	TMS980581_D1	17580533	15733013	89,5	70,9	1,16%	1318992	7,5
D4_11	TMS980581_D1	13290345	11765451	88,5	70,89	1,14%	934711	7,0
D4_13	TMS980581_R2	13203675	12467995	94,4	72,27	0,98%	627365	4,8
D4_15	TMS980581_R2	9997368	8909244	89,1	70,87	1,02%	785512	7,9
D4_17	TMS980581_R2	15738358	13486291	85,7	69,09	1,01%	1638969	10,4
D4_19	TMS980581_R3	10869231	9843831	90,6	71,01	1,02%	802693	7,4
D4_21	TMS980581_R3	11682925	10638423	91,1	70,7	1,03%	836989	7,2
D4_23	TMS980581_R3	11147532	10005775	89,8	69,76	1,03%	910290	8,2
E4_1	TMS30572_R1	18868401	16969726	89,9	70,9	1,25%	1496428	7,9

E4_3	TMS30572_R1	19873742	17724288	89,2	69,99	1,28%	1523876	7,7
E4_5	TMS30572_R1	22178919	19438863	87,6	70,68	1,23%	1619406	7,3
E4_7	TMS30572_D1	18048481	14689746	81,4	69,89	1,26%	1396581	7,7
E4_9	TMS30572_D1	23884983	21238781	88,9	70,44	1,25%	1825388	7,6
E4_11	TMS30572_D1	11014150	9908389	90,0	70,47	1,27%	764569	6,9
E4_13	TMS30572_R2	12079812	11208869	92,8	72	1,07%	657190	5,4
E4_15	TMS30572_R2	9433100	8579262	90,9	70,98	1,08%	667467	7,1
E4_17	TMS30572_R2	11608204	10338084	89,1	69,91	1,20%	878974	7,6
E4_19	TMS30572_R3	15199728	13497810	88,8	70,09	1,09%	1220256	8,0
E4_21	TMS30572_R3	9955003	8965990	90,1	70,14	1,12%	801289	8,0
E4_23	TMS30572_R3	10484348	9477994	90,4	70,21	1,12%	843341	8,0

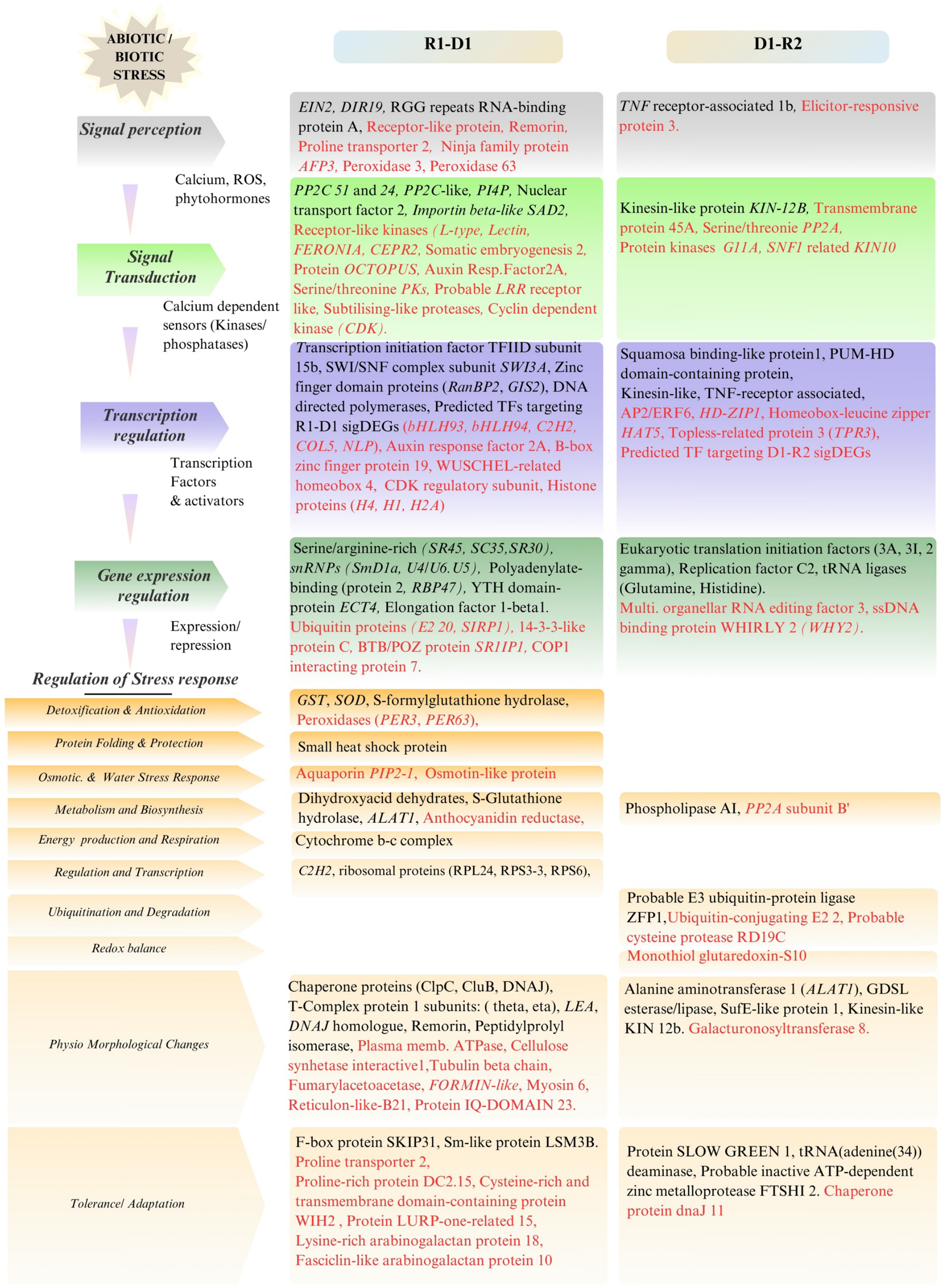

Figure S1. Stress Signal Perception to Response Cascade in Cassava During Seasonal Transitions. This figure illustrates the cascade of reactions involved in stress perception and response, leading to adaptation and tolerance in plants (left side). It highlights cascade elements found in the shared transcriptome profiles of four cassava genotypes, comparing transitions from the rainy season to the dry season (R1-D1) and from the dry season to the rainy season (D1-R2). Upregulated genes are shown in black, while downregulated genes are depicted in red.
